# Aligning bladder cancer research with patient needs: an update on research priorities

**DOI:** 10.1111/bju.70206

**Published:** 2026-02-27

**Authors:** Nada Humayun‐Zakaria, Benjamin J. Tura, Mieke van Hemelrijck, Richard T. Bryan

**Affiliations:** ^1^ Bladder Cancer Research Centre University of Birmingham Birmingham UK; ^2^ Transforming OUtcomes through Research King's College London London UK

**Keywords:** patient‐centred research, bladder cancer, non‐muscle‐invasive bladder cancer, muscle‐invasive bladder cancer, biomarkers, surveillance

**Abbreviations**
MIBC: muscle‐invasive bladder cancer
NMIBC: non‐muscle invasive bladder cancer

Bladder cancer is associated with substantial morbidity and long‐term surveillance burden, particularly for patients with non‐muscle‐invasive disease, with significant impact on quality of life. In 2019, Bessa et al. [1] published the first stakeholder‐driven (patients and healthcare professionals) consensus outlining the top 10 research priorities in bladder cancer. These 10 research priorities could be categorised into three research themes: predictive and prognostic biomarkers, optimisation of diagnostic pathways, and surveillance following radical therapy [1]. Their work represented a landmark shift towards the systematic integration of patient, clinical, and scientific perspectives in shaping the research agenda. Recognising rapid advancements in genomic biomarker discovery [2], molecular profiling [3], and novel therapeutics [4] in the years since, notwithstanding the effects of the global pandemic, we conducted an updated consensus exercise to reassess contemporary research needs.

Using an interactive survey methodology adapted from the original study, we engaged 27 individuals including healthcare professionals, researchers across a spectrum of disciplines, charities, and patient advocates in live polling and facilitated discussion, evidence review, and iterative ranking of pertinent research questions. Rankings from the updated exercise were compared with those from 2019 to assess changes in emphasis over time.

This updated prioritisation highlights an ongoing shift towards a biomarker‐led, precision‐medicine research agenda in bladder cancer. While not intended to define clinical standards, the findings provide direction for future translational studies, biomarker validation efforts, and trial design, with relevance to the risk‐adapted management of both non‐muscle‐invasive bladder cancer (NMIBC) and muscle‐invasive bladder cancer (MIBC).

The most highly ranked research question focused on whether validated urinary biomarkers could substitute or reduce the frequency of cystoscopy follow‐up in patients with high‐risk NMIBC. The burden of frequent cystoscopy (citing discomfort, anxiety, procedural risks) and the cumulative resource impact on healthcare systems remains an issue. Compared with the Bessa et al. [1] findings, the strength of preference for non‐invasive approaches has intensified, reflecting increasing patient awareness of emerging biomarker technologies.

Eight of the top 10 priorities (compared to six in 2019) directly involved biomarker development, validation, or implementation. These include biomarkers for NMIBC surveillance and metastatic disease, diagnostic markers to improve early detection, molecular profiling and genomic stratification of NMIBC and MIBC, predictive biomarkers for neoadjuvant chemotherapy and immunotherapy, and biomarkers supporting early cystectomy decision‐making. This biomarker‐driven vision demonstrates an enhanced translational focus within the bladder cancer community and aligns strongly with contemporary precision‐medicine frameworks [5, 6].

Patient advocacy input continues to indicate emphasis on quality‐of‐life considerations including reduced invasiveness, acceptability of tests, and minimisation of procedure‐related burden. These values influence the importance of non‐invasive diagnostics, early detection, and timely, personalised treatment strategies. Similar to the 2019 review, patient perspectives helped ensure that priorities reflected real‐world needs rather than solely technological potential.

Several newly elevated priorities reflect the rapid evolution of therapy and molecular oncology for risk stratification (Table 1). Priorities focused on therapy include predictive biomarkers for neoadjuvant therapy (recognising the heterogeneous response to chemotherapy and immunotherapy) and integration of molecular profiling (particularly to guide treatment selection in high‐risk NMIBC and MIBC). Considerations around risk include genomic risk stratification and enabling identification of patients at high risk of progression. These themes collectively indicate increasing integration of genomic and molecular tools into clinical decision‐making, signalling a maturation of precision urology.

**Table 1:** Comparison of the top research priorities in bladder cancer between the 2025 and 2019 stakeholder consensus exercises. Items denoted as N/A in 2025 were included and ranked in the 2019 consensus but did not feature in the updated prioritisation. Two additional research questions were introduced and ranked in the 2025 exercise which were not part of the 2019 consensus.

Research question	2025 ranking	2019 ranking
In high‐risk NMIBC, could frequency of cystoscopy follow‐up be substituted with urinary tests to improve patient acceptability and reduce costs without increasing the risk of progression?	1	N/A
Can new urinary and/or blood‐based markers improve the detection of BC recurrence after treatment and/or detection of metastatic BC?	2	7
Can new urinary and/or blood‐based markers replace invasive techniques in the diagnosis of BC?	3	5
Following definitive treatment for MIBC, what is the optimal strategy for surveillance?	4	9
Which biomarkers would allow us to better select high‐risk NMIBC patients for early cystectomy?	5	4
Could predictive biomarkers improve outcomes in advanced BC, particularly in neoadjuvant therapy?	6	N/A
Could molecular profiling of MIBC help select and stratify patients for treatments with respect to the risk of relapse and/or prognosis?	7	1
Which prognostic biomarkers are useful in patients with high‐risk MIBC and NMIBC to select effective radical treatments?	8	2
Can new genomic markers help us risk stratify patients and predict response to therapies?	9	3
Is there a role for immune checkpoint inhibitors in combination with chemotherapy in the neoadjuvant setting, particularly in advanced BC?	10	N/A
Can new validated technologies improve the detection of metastatic disease in regional lymphatics and distant sites?	N/A	6
Which PET imaging agent/ tracer would increase the sensitivity and specificity of metastatic BC detection?	N/A	8
What is the consequence of delay between TURBT and definitive treatment?	N/A	10

BC, bladder cancer; PET, positron emission topography; TURBT, transurethral resection of bladder tumour.

The priority areas identified have both immediate and long‐term implications for clinical practice. Immediate benefits from translational innovations that are available now include reduced cystoscopy burden, improved patient experience and acceptability, and potential cost reductions for healthcare systems [2]. Long‐term benefits include personalised treatment pathways informed by molecular features and/or liquid biopsies [2, 3, 7], improved survival through refined therapy selection, and enhanced quality of life via minimisation of invasive procedures.

The consensus also underscores the need for multicentre biomarker validation studies, real‐world implementation research, and integration of genomic, transcriptomic and liquid biopsy data into risk‐stratified clinical pathways. Stakeholders highlighted the importance of robust evidence generation, pragmatic study design, and widespread adoption of quality‐of‐life metrics in future trials.

Overall, this updated exercise confirms the enduring relevance of the original priorities while demonstrating an evolution towards a biomarker‐led and molecularly‐profiled personalised approach to bladder cancer research. The prioritised themes provide a clear roadmap for clinicians, researchers, funders, and policymakers to reduce the burden of bladder cancer. Continued stakeholder engagement, dedicated funding for biomarker validation, and coordinated multicentre collaboration will be essential for translating these priorities into improved patient outcomes.

## Disclosure of Interests

Richard T. Bryan is an unpaid charity trustee for Action Bladder Cancer UK (UK), an advisory board member for Nonacus Limited (UK), and receives consultancy fees from Cystotech ApS (Denmark) and Nonacus Limited (UK). The remaining authors have no disclosures.
